# Polymorphism of The Regulatory Region of the *ITGAM* Gene (-323G>A) as a Novel Predictor of a Poor Nutritional Status in Head and Neck Cancer Patients Subjected to Intensity-Modulated Radiation Therapy

**DOI:** 10.3390/jcm9124041

**Published:** 2020-12-14

**Authors:** Marcin Mazurek, Radosław Mlak, Iwona Homa-Mlak, Tomasz Powrózek, Anna Brzozowska, Paweł Gołębiowski, Teresa Małecka-Massalska

**Affiliations:** 1 Department of Human Physiology, Medical University of Lublin, 20-059 Lublin, Poland; radoslaw.mlak@gmail.com (R.M.); iwona.homa.mlak@gmail.com (I.H.-M.); tomasz.powrozek@gmail.com (T.P.); teresa.malecka-massalska@umlub.pl (T.M.-M.); 2 II Department of Radiotherapy, Center of Oncology of the Lublin Region St. John of Dukla, 20-059 Lublin, Poland; annabrzo@poczta.onet.pl (A.B.); golebiowski.pawel@wp.pl (P.G.)

**Keywords:** head and neck cancer, cancer cachexia, malnutrition, polymorphism, radiotherapy, IMRT, *ITGAM*

## Abstract

Background: The most serious disturbance of the nutritional status is neoplastic cachexia. The main factor contributing to the development of cachexia is the ongoing inflammatory process. The gene associated with the development of the inflammatory response is *ITGAM*. Therefore, the aim of the study was to assess the relationship between a single nucleotide polymorphism (SNP)-323G>A of the *ITGAM* gene and the occurrence of nutritional disorders in patients undergoing radiotherapy (RT) due to head and neck cancers (HNC). Methods: The study involved 71 patients with HNC treated with intensity-modulated radiotherapy (IMRT). SNP analysis of the *ITGAM* gene (-323G>A) was performed using commercial molecular probes and Real-Time PCR. Results: The presence of the A allele of the *ITGAM* gene significantly (over 14-fold) reduced the risk of severe disturbances in nutritional status assessed according to the subjective global assessment (SGA) scale (odds ratio (OR) = 0.07; *p* = 0.0213). The GG genotype of this gene was associated with an over three-fold higher risk of shortened overall survival (OR = 3.01; *p* = 0.0376). Conclusions: Determination of the SNP (-323G>A) of the *ITGAM* gene may prove to be a useful marker in the assessment of the risk of nutritional disorders in patients with HNC undergoing RT.

## 1. Introduction

Head and neck cancers (HNC) are mainly located in the pharynx, nasopharynx, larynx and oral cavity. Smoking, alcohol abuse and, in the case of oropharyngeal cancers, human papillomavirus (HPV) infection are considered to be the main risk factors for developing HNC [1]. According to epidemiological data, approximately 630,000 new cases of HNC are diagnosed annually, and the most common histological form is squamous cell carcinoma, accounting for 90% of cases [1]. In recent years, developing countries have seen a nearly two-thirds increase in the incidence of HNC [2]. Worldwide, 350,000 people die from HNC every year [1].

HNC treatment is based primarily on the use of surgery, radiotherapy (RT), chemotherapy (CTH) and chemoradiotherapy (C-RT) depending on the stage of cancer progression. However, since most patients are diagnosed when the cancer is at an advanced stage, RT is the axis of treatment in clinical practice. More and more often, in HNC therapy, traditional RT is abandoned and its modifications (e.g., intensity-modulated radiotherapy, IMRT) are used in order to reduce the side effects of the therapy. In the case of IMRT, tumor tissue is exposed to sufficiently high doses of ionizing radiation. Therefore, in the case of sensitive tissues, especially in the area of the head and neck (brain stem, spinal cord, parotid glands, and optic nerve), the use of IMRT contributes to increasing safety and shortening the treatment time [3,4,5].

More precise radiation dose targeting at the tumor tissue while protecting healthy tissues reduces the risk of early and late toxicity symptoms: oral mucositis (OM), dysphagia, and xerostomia [6]. The onset of dysphagia symptoms is observed already with a dose of 50–60 Gy [6]. The use of IMRT contributes to the reduction of the risk of the development of OM, but does not rule out the occurrence of other toxicities [7]. Malnutrition symptoms arising during RT or C-RT treatment occur in 44–88% of patients. Critical weight loss (CWL) is much more common in patients with HNC undergoing RT (>5% during RT and >7.5% by week 12 following treatment). In addition, the development of CWL is associated with poor long-term prognosis [8]. The use of IMRT alone translates into a reduction in the percentage of patients who require a “feeding tube” compared to patients with HNC treated with three-dimensional conformal radiation therapy (3DC-RT) (49% vs. 72%). A similar relationship is observed in the group of patients treated with chemo-IMRT compared to 3DC-RT (63% vs. 82%) [9]. Cancer cachexia has a multifactorial basis; it is characterized by the predominance of catabolic processes, which result in the progressive loss of fat and muscle tissue. It can develop to the subsequent, irreversible stage of malnutrition [8,10]. It is suspected that the main mechanism responsible for the development of neoplastic cachexia is the increased inflammation manifested by an increase in pro-inflammatory cytokines: TNFα, INF-γ and IL-6. When tissues are exposed to a beam of ionizing radiation, the NFκ-B pathway activation occurs, the production of cytokines responsible for the induction of inflammation and the generation of large amounts of free radicals. The resulting cytokines participate in the pathways activating the processes related to the destruction of muscle fibers as well as the reduction of adipose tissue [8]. IL-6 participates in the development of inflammation in the course of many cancers: breast, prostate, colon, lung, stomach and brain [11]. The overproduction of IL-6 contributes to the promotion of processes conducive to the breakdown of muscle tissue (attempts have been made to use this parameter to monitor the nutritional status of the patient), but it is worth noting that in some cases, cachexia may develop without a marked increase in typical pro-inflammatory cytokines (including IL-6) [12,13]. This prompts the search for other markers specific to HNC-related eating disorders. In the case of patients with HNC, it was found that nutritional disorders contribute to a worse response to treatment, deterioration of the quality of life, toxic effects of therapy, longer hospitalization and shorter survival time [8]. This is an important premise for the search and determination of prognostic factors for patients with nutritional disorders.

One of the genes associated with the development of the inflammatory response is *ITGAM,* also known as CD11b, Mac-1 integrin alpha chain or complement receptor 3, located on the 16p11.2 chromosome [14]. The protein product of the *ITGAM* gene is responsible for the functioning of INF-γ receptors and the regulation of the secretion of inflammatory mediators. When ITGAM expression is low on the surface of antigen-presenting cells (APCs), interleukin (IL) production is increased, including IL-6 and IL-17 [14,15]. The effect of single nucleotide polymorphism (SNP) rs114367 on the level of both the ITGAM transcript and protein on monocytes’ surface in patients with systemic lupus erythematosus (SLE) has been demonstrated. The mRNA level depended on the genotype (and the amount of protein was proportional to the level of the transcript). More than a two-fold decrease in protein levels occurred in patients with AA genotype (predisposing them to higher risk) compared to patients with GG genotype (lower risk group). Differences in the expression of this protein may result from the allele-specific decrease in transcription repression [16]. However, to our knowledge, there are no studies on the correlation between the *ITGAM* gene status and the development of nutritional disorders in patients with HNC. The aim of this study was to assess the relationship between SNPs located in the regulatory region of the *ITGAM* gene (-323G>A) and the occurrence of nutritional disorders in patients with HNC undergoing IMRT.

## 2. Materials and Methods

### 2.1. Study Group

69 patients diagnosed with advanced HNC and treated at the Department of Oncology of the Medical University of Lublin in 2014–2017 were qualified for the study. The following inclusion criteria were used: age over 18, both women and men, and histopathologically confirmed head and neck cancer.

The study included patients with histologically confirmed advanced HNC in stage III (28.98%) or IV (71.02%) according to the TNM classification. The median age of patients was 63 years (range: 42–87 years). Men predominated in the study group (85.51%). HNC was located in the larynx in 52.17% of patients and in the nasopharynx in 42.03%. A total of 42.03% of patients were treated with surgery and RT, 26.09% were treated with surgery and C-RT, 13.04% were treated only with RT, 4.35% received induced CTH with RT and 8.69% were treated with C-RT. Treatment with the induced CTH method and C-RT was used in 4.35% of patients, and in the case of 1.45%, this method was supplemented with surgery. The decision to implement parenteral nutrition was made in 11.59% of patients. 63.77% of the subjects experienced critical weight loss (CWL). The study group characteristics are presented in Table 1.

### 2.2. IMRT

Patients undergoing IMRT were subsequently included in the study group (patients could have previously undergone surgery and/or CTH). On the other hand, people diagnosed with other types of cancer (melanoma, lymphoma) and previous cancer cases, including HNC located in the RT site, were excluded from the study. The Eastern Cooperative Oncology Group (ECOG) scale was used to assess the patient’s physical performance level. Alcohol consumption was assessed according to the International Statistical Classification of Disease and Related Health Problems (ICD). The nutrition risk screening (NRS) scale was used to assess patients’ nutritional risk, while the subjective global assessment (SGA) was used to assess the degree of nutrition. Based on the SGA scale, patients were divided into those with good nutritional status (SGA-A) and the ones with moderate (SGA-B) or severe (SGA-C) malnutrition. Based on the work of Langius et al., critical weight loss (CWL) was defined as a weight loss of >5% from the start of RT to week 4 or >6.25% to week 7 of RT [17]. The following groups were distinguished among smokers: former and current smokers. A non-smoker is a person who has never smoked tobacco or a person who has smoked less than 100 cigarettes in their lifetime. The current smoker or ex-smoker is defined as an adult who has smoked at least 100 cigarettes during his life or is still smoking. The treatment was based on the IMRT technique using ONCOR (Siemens, München, Germany) linear accelerator at the dose of 54–70 Gy (the daily dose was 2 Gy). In patients in advanced stages, a total dose of 70 Gy was used in 35 fractions per tumor and enlarged lymph nodes. The 66 Gy irradiation dose in 33 fractions was received by patients with high volume risk who underwent surgery. In contrast, patients with an average and low risk received 60 and 54 Gy, respectively. Doses of 54 Gy or 60 Gy were used to treat elective lymph nodes. In some patients, in addition to irradiation, treatment with cisplatin and 5-fluorouracil (1–4 cycles of chemotherapy) was used.

### 2.3. Genotyping

Prior to the commencement of the study, 5 mL of peripheral blood were collected from each person participating in the study and stored at −80 °C until laboratory analysis. DNA isolation was performed using the column method with a dedicated kit according to the manufacturer’s recommendations (DNA Blood Mini Kit, Quiagen, Canada). Subsequently, the spectrophotometric evaluation of the concentration and quality of the obtained DNA was performed using the NanoDrop Lite Spectrophotometer (Thermo Fisher Scientific, Waltham, MA, USA). The genotyping reaction based on the Real-Time PCR technique was performed on a StepOnePlus device (Applied Biosystems, Foster City, CA, USA) according to the manufacturer’s protocol using Genotyping Master Mix and TaqMan probes specific for *ITGAM* SNP (rs7193943) (Thermo Fisher Scientific, Waltham, MA, USA). For genotyping, we chose the Real-Time PCR method, which is commonly used both in scientific research and in routine genetic diagnostics (many tests have the necessary diagnostic certification) and the necessary probes are commercially available, which makes the results obtained by different research teams comparable. The qPCR was performed in 10 µL reaction volume on 96-well plates. To each well reaction mix containing 5 µL of Taqman Genotyping Master Mix, 0.5 µL of Taqman SNP assay (20×) and 4.5 µL 0.2 ng/µL diluted DNA template were added. The thermal cycling conditions consisted of enzyme activation at 95 °C, 40 cycles of denaturation at 95 °C for 15 s and annealing at 60 °C for 1 min. All sample tests were performed in triplicates. After amplification, the genotypes variants were obtained and analyzed on StepOne Software v2.3 (Applied Biosystems, Foster City, CA, USA). In addition, 10% randomly selected samples were re-analyzed by sequencing technology (3500 Genetic Analyzer, Life Technologies, Carlsbad, CA, USA); results are 100% consistent. The manufacturer has validated the selected probe.

### 2.4. Statistical Analysis

The statistical analysis of the results was performed using the MedCalc 12.7 software (MedCalc Software, Belgium). Results of *p* < 0.05 were considered statistically significant. In order to assess the risk of malnutrition (SGA), nutritional risk (NRS) and CWL depending on demographic, clinical and genetic factors, an analysis was performed using the odds ratio (OR) test with 95% Confidence Interval (95% CI). Univariate overall survival (OS) analysis was performed with the use of the two-side log-rank test (with the calculation of the risk coefficient–hazard ratio, HR) and visualized with the use of Kaplan–Meier estimation, whereas Cox logistic regression models were used in multivariate OS analysis with statistically significant factors from univariate analysis (α < 0.05) as included variables. Comparisons of the distribution of demographic and (continuous) nutritional variables depending on the genotype of the studied gene, nutritional status assessed by SGA, nutritional risk (NRS scale) and the occurrence of CWL were performed using the non-parametric Mann–Whitney U test.

## 3. Results

### 3.1. Factors Affecting the Risk of Malnutrition According to SGA Scale

In the group diagnosed with non-squamous cell carcinoma, there was a 6.67-fold lower risk of moderate and severe malnutrition (SGA) approaching significance (OR = 0.15; *p* = 0.0530). According to the SGA scale, there was a significant 5.5-fold higher risk of moderate and severe malnutrition in the non-oropharyngeal group (OR = 5.50; *p* = 0.0089). In patients with cancer located outside the larynx, a significant, 4-fold lower risk of moderate and severe malnutrition was found according to SGA scale compared to other locations (OR = 0.25; *p* = 0.0319). A 48-fold higher risk of a diagnosis of moderate and severe disturbances in nutritional status was noted in the case of people with the T4 trait (OR = 48.30; *p* = 0.0080). The risk of moderate or severe malnutrition significantly higher than two-fold was observed in non-smokers (OR = 2.29; *p* = 0.0466). Moreover, the risk of moderate or severe malnutrition significantly higher than 5.5-fold was observed in patients with allele G (OR = 5.50; *p* = 0.0089).

According to the SGA scale, more than 4.5 times higher risk of severe malnutrition was observed in patients with stage T4 neoplasm (OR = 4.71; *p* = 0.0063). The G allele presence was associated with a significantly higher (approximately 31-fold) risk of severe malnutrition (OR = 30.95; *p* = 0.0013). On the other hand, the presence of the A allele significantly reduced (more than 14-fold) the risk of severe disturbances in the nutritional status (OR = 0.07; *p* = 0.0213) (Table 2).

### 3.2. Factors Affecting the Nutritional Risk According to NRS Scale

None of the examined factors (demographic, clinical, genetic) had a statistically significant influence on the risk of a higher degree (≥3) of nutritional risk according to the NRS scale (Table 3).

### 3.3. Factors Affecting the Risk of CWL

The tumor localization other than the nasopharynx was associated with an approximately 11-fold lower risk of CWL (OR = 0.09; *p* = 0.0001). Furthermore, patients with the A allele had an approximately 14-fold lower risk of developing CWL (OR = 14; *p* < 0.0001). Patients with the A allele had an approximately 11-fold lower risk of developing CWL (OR = 0.09; *p* = 0.0353) (Table 4).

### 3.4. Overall Survival

**Figure 1:**
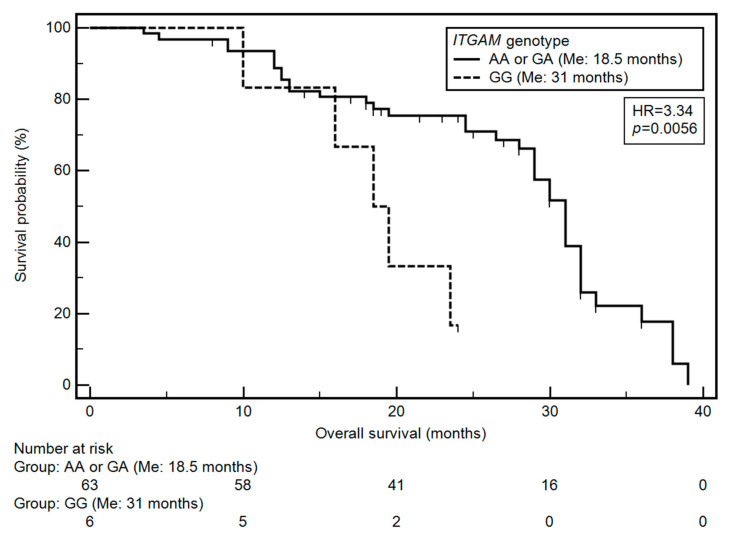
Impact of ITGAM on patients’ overall survival: differences in overall survival between groups of patients with GG and AA or GA genotype.

Based on the univariate and multivariate analysis (after considering age, M classification, TNM classification and *ITGAM* genotype), it was found that only the TNM classification and the *ITGAM* gene SNP had a significant effect on OS. Grade IV HNC was significantly associated with a higher risk of a shorter OS (HR = 4.14; *p* = 0.0135). On the other hand, the GG genotype of *ITGAM* gene was associated with an over three-fold higher risk of shortened overall survival (OR = 3.34, *p* = 0.0056; Figure 1). The results of the univariate and multivariate analysis are presented in Table 5.

### 3.5. Comparisons of Demographic, Clinical and Nutritional Variables According to ITGAM Genotypes

Significantly higher albumin concentration was observed in patients with AA genotype, compared to GA or GG genotype carriers (3.4 vs. 3.3 g/l; *p* = 0.0241). Whereas significantly lower values were observed in patients with the GG genotype as compared to the carriers of the AA or GA genotypes (3.1 vs. 3.3 g/l; *p* = 0.0268) (Supplementary Table S1).

### 3.6. Comparisons of Demographic, Clinical and Nutritional Variables According to SGA

Patients with good nutritional status had significantly higher body weight compared to patients with moderate or severe malnutrition (SGA A vs. B or C) (76.0 vs. 62.0 kg; *p* = 0.002). In patients with normal nutritional status, significantly higher Body Mass Index (BMI) values were observed compared to patients with moderate or severe malnutrition (SGA A vs. B or C) (25.0 vs. 21.5 kg/m^2^; *p* = 0.0001). In patients with good nutritional status, significantly higher albumin values were noted compared to subjects with moderate or severe malnutrition (SGA A vs. B or C) (3.8 vs. 3.3 g/l; *p* < 0.0001). In the group of patients with moderate or severe malnutrition, the value of the Normalized Fat-Free Mass Index (nFFMI) index was lower than in the group of patients with normal nutritional status (SGA B or C vs. A) (16.4 vs. 17.4 kg/m^2^; *p* < 0.0275). In patients with normal nutritional status and with moderate malnutrition, a significantly higher BMI was demonstrated compared to the group with severe malnutrition (SGA A or B vs. C) (24.4 vs. 21.2 kg/m^2^; *p* < 0.0234). In the group of patients without malnutrition and with moderate malnutrition, the concentration of albumin was significantly higher than in patients with severe malnutrition (SGA A vs. B or C) (3.4 vs. 3.2 g/l; *p* < 0.0011) (Supplementary Table S2).

### 3.7. Comparisons of Demographic, Clinical and Nutritional Variables According to NRS

Patients at low risk of being subjected to nutritional treatment had a higher body weight compared to patients at high risk of getting nutritional treatment (NRS < 3 vs. ≥3) (68.0 vs. 59.5 kg; *p* < 0.0208). Patients at low risk of being subjected to nutritional treatment had a higher BMI compared to patients at high risk of getting nutritional treatment (NRS < 3 vs. ≥3) (23.1 vs. 19.7 kg/m^2^; *p* < 0.0026). In the group of patients at low risk of receiving nutritional treatment, the FFMI index was significantly higher than in patients with high risk of getting nutritional treatment (NRS < 3 vs. ≥3) (16.7 vs. 16.1 kg/m^2^; *p* < 0.0353). The nutritional value of the nFFMI index was significantly higher compared to patients at high risk of being subjected to nutritional treatment (NRS < 3 vs. ≥3) (17.4 vs. 16.5 kg/m^2^; *p* < 0.0291) (Supplementary Table S3).

### 3.8. Comparisons of Demographic, Clinical and Nutritional Variables According to CWL

The group of patients with CWL showed significantly lower body weight compared to those without CWL (61.5 vs. 69.0 kg; *p* = 0.0400). A significantly lower value of the FFM index was also demonstrated in patients with CWL (45.6 vs. 53.1 kg/m^2^; *p* = 0.0123). In the case of CWL patients, significantly lower FFMI values were demonstrated (16.5 vs. 17.3 kg/m^2^; *p* = 0.0206). Patients with CWL showed a significantly lower value of the nFFMI parameter compared to patients without critical weight loss (17.1 vs. 18.5 kg/m^2^; *p* = 0.0425) (Supplementary Table S3).

## 4. Discussion

A characteristic process associated with neoplastic disease development is malnutrition, which often turns into cancerous cachexia. This condition is associated primarily with a significant loss of muscle mass and adipose tissue. Cancer cachexia is usually irreversible even after nutritional treatment has been instituted and is the leading cause of death in the group of patients diagnosed with cancer [1,2,3,4]. Additional symptoms that are characteristic of neoplastic cachexia are emerging anemia, anorexia, weakness and deterioration of the patient’s mental state. The treatment used in patients diagnosed is less effective and in the case of radiotherapy or chemotherapy is associated with a higher risk of adverse effects [6,7]. This disease is characteristic of cancers of the lung, liver or the head and neck region. Symptoms of cancerous cachexia are diagnosed in 52% of patients with advanced-stage HNC disease. If the available cancer treatment is used: RT, CTH or C-RT, this percentage ranges from 44% to 88% in patients with HNC [18,19]. The pathomechanism of neoplastic cachexia is based mainly on the increased demand of the body’s cells for energy, leading to the predominance of catabolic processes while reducing appetite. The development of inflammation has a great influence on the mechanism of the development of nutritional disorders [1,2,3,4]. Then, the following inflammatory cytokines increase the increase of production: INF-γ, TNF-α, IL-1β and IL-6. These mediators have been found to be involved in the mechanisms responsible for the breakdown of muscle fibers in the Ubiquitin-Proteasome Pathway (UPP) by activating the muscle atrophy F box (MAFbx) ligase and muscle ring-finger 1 (MuRF1) genes. Inflammation has also been confirmed to be important in the breakdown of adipose tissue. Increased production of TNF-α is associated with an increase in the number of fat cells undergoing apoptosis and a decrease in the rate of lipogenesis.

Interacting with IL-1 reduces the transport of glucose and free fatty acids into cells, which is associated with reduced synthesis of adipose tissue. IL-6 is also involved in the catabolism of both adipose tissue and muscle tissue. Determining the level of this interleukin may be of great importance in assessing the nutritional status and further prognosis [8,14,15].

Treatment and other clinical or genetic factors may contribute to weight loss in patients with HNC. In the case of RT, i.a., Baseline BMI, tumor location, histopathological type, age, or gender may cause differences in the level of weight loss in individual patients. CWL usually develops in patients treated with C-RT, exposed to higher doses of ionizing radiation (>65 Gy) [20]. CWL is also found at BMI > 25 (OR: 3.00; *p* < 0.0001), more often in patients with advanced HNC (T3-T4) (OR: 1.68; *p* = 0.0300) and with cancer of the throat or mouth (OR: 3.12; *p* < 0.0001) [21].

The main task of the *ITGAM* gene is coding the αMβ2-integrin chain. Therefore, it is of key importance in the activation of leukocytes, their migration and adhesion. CD11b is involved in the regulation of B-cell signaling and the function of INF-γ receptors, Toll-like receptors, B-cell receptors and Fcγ receptors. This molecule is essential in the pathomechanism of autoimmune diseases, most often SLE [14]. *ITGAM* may influence the production of inflammatory cytokines. High expression of *ITGAM* is observed on APCs. In the case of low expression of *ITGAM* on the surface of APCs, there is an increased production of IL-6 and, as a consequence, activation of T lymphocytes and an increase in the production of IL-17 [15]. A similar correlation has been found in the case of the increased production of the other pro-inflammatory factors, i.e., IL-1β, TNF α and IFN β [22].

The increased levels of IL-6 contribute to the increased activation of the JAK/STAT pathway, especially the STAT-3 domain, which is involved in the breakdown of muscle fibers. The involvement of IL-6 in STAT-3 signaling and its participation in sarcopenia development translates into increased weight loss. Additionally, this mechanism contributes to the development of malnutrition and cachexia in patients diagnosed with cancer. The study by GuneyEskiler et al. involved 18 patients with lung, stomach or breast cancer with concomitant neoplastic cachexia, 30 patients without accompanying symptoms of cachexia and 25 healthy people. A higher IL-6 gene expression level was confirmed in patients with cachexia developing during the studied neoplasms compared to the group without cachexia and healthy people. A similar correlation was noted in the case of transcription factor STAT-3 expression in the group of patients with breast, lung or stomach cancer with neoplastic cachexia compared to patients with these types of neoplasms without cachexia and healthy people [11]. Another study assessed the level of IL-6 (protein) in serum samples of 94 people with cachexia and in a group of 16 people not diagnosed with cachexia. Higher levels of IL-6, IL-8, IL-1β and TNFα have been demonstrated in patients with confirmed cachexia. There was also a positive correlation between IL-6 levels and weight loss [23]. In another study, 26 male BALB/c mice had colon cancer cells injected subcutaneously to induce neoplastic disease, causing the development of severe cachexia. In some of the mice, subcutaneous injections of antibodies against the IL-6 receptor were performed, and the control group consisted of mice subcutaneously injected with saline. The study assessed the level of IL-6 and the level of free fatty acids (FFA) to assess the effect of this cytokine on the breakdown of adipose tissue in the initial and advanced stages of cancer. Increased IL-6 and FFA levels were noted in mice with developing neoplasm, both in the early and late stages. On the other hand, in mice with anti-IL-6 receptor antibodies, inhibition of cytokine-dependent lipolysis and browning of adipose tissue was observed. Additionally, an increase in the expression of genes responsible for the decay of the carcass tissue was found in cachexia’s initial phase (Hsl, Cgi58 and Atgl) [24]. In addition to Il-6, other cytokines play a significant role in disturbing the metabolism of adipose tissue, thus determining both the reduction of fatty acid production (IL-1) and adipocyte apoptosis (TNF-α) [25].

The study by Zhang et al. concerned the role of SNP -634C>G of the *IL-6* gene in a group of 128 patients diagnosed with pancreatic cancer in the assessment of cancer cachexia. Cachexia was confirmed in 67.46% of the patients. A significantly more frequent cachexia occurrence was found in carriers of the G allele (20% vs. 8%, *p* = 0.021). Moreover, OS in patients with GG and CG genotypes was significantly shorter than in patients with CC variant of this gene (*p* = 0.023). However, no significant correlation was confirmed between the -634C>G polymorphism of the *IL-6* gene and other demographic and clinical factors (e.g., age, weight, degree of advancement or C Reactive Protein (CRP) level). The above study did not show a significant correlation between the other tested IL-6 SNPs and the occurrence of cachexia in the course of pancreatic cancer [26].

In the study by Powrózek et al., SNP for TNF-α –1031T/C (rs 1799964) was determined in a group of 62 patients with HNC undergoing RT. Patients with the CC genotype had a significantly higher risk of cachexia (OR = 3.724 *p* = 0.019). According to the SGA scale, significantly higher risk of disturbances in nutritional status occurred in patients with the C allele (CC and CT) compared to TT homozygotes (OR = 13.29; *p* = 0.0001). A significant decrease in body weight was found in patients with the CC genotype (*p* = 0.045) compared to the other variants. In patients with the CC genotype, a significant increase in serum TNF-α level (*p* = 0.006) and a decrease in total protein level (*p* = 0.044) were confirmed. Moreover, the authors showed a significant correlation between the C allele’s presence and an increased risk of developing nutritional disorders, including cachexia, in patients with HNC (OR = 9.737; *p* = 0.044). For CC homozygotes, a 38-fold increase in the risk of qualifying for grade 3 or 4 risk of malnutrition on the NRS scale was demonstrated (*p* = 0.015). The same genotype also determined the shortening of the OS compared to the CT and TT genotypes (28 vs. 38 months, HR = 3.63; *p* = 0.013) [27].

In the case of determining the SNP for SELP - 2028 C/T (rs3917647), the study group consisted of 66 people diagnosed with and treated for HNC. According to the SGA scale, HNC patients with the CC genotype had a significantly higher risk of severe malnutrition (OR = 4.04, *p* = 0.015). In patients with the CC genotype receiving parenteral nutrition, a higher risk of low BMI values (<18.5) (OR = 39, *p* = 0.036) was observed and a three-fold higher risk of shortening the survival time (29 vs.34 months, HR = 3.02, *p* = 0. 0085) compared to patients with other SNP variants of the studied gene. However, a four-fold lower risk of malnutrition was noted in TT homozygotes (OR = 0.24, *p* = 0.048). There was also a significantly higher total protein level assessed before the initiation of treatment in TT homozygotes for the *SELP* gene (6.68 vs. 6.26 g/l, *p* = 0.030) [28].

It should be noted that the available literature lacks studies related to the relationship between *ITGAM* gene status and nutritional disorders. The available studies focus mainly on this gene SNP’s role in the course of diseases in the rheumatological conditions-mainly SLE and lupus nephritis (LN) [14,22].

Searching for new markers of inflammation involved in the development of malnutrition and cancer cachexia and explaining their action mechanisms may allow for a more efficient diagnosis of nutritional disorders, faster implementation of nutritional treatment, and the development of new therapies. The presence of the studied SNP in the regulatory region of the *ITGAM* gene may significantly impact the expression of this gene and, consequently, the production of a specific protein. This may translate into regulating the production of pro-inflammatory cytokines such as IL-6. Therefore, the chronic inflammation developing against the background of increased cytokine production, characteristic of patients with HNC undergoing RT, may result in disorders in the nutritional status and, consequently, may lead to irreversible changes cachexia.

Our study’s limitations include the lack of assessment of the influence of diet or emerging problems with food intake caused by the presence of the tumor itself (dysphagia) on the development of nutritional disorders. Moreover, we did not assess the influence of individual genotypes of the studied gene on its expression (and the expression of the protein it encodes). Another limitation is the small size of the study group and the fact that apart from RT treatment, which included all patients, some of them underwent prior treatment (surgical treatment, C-RT). Despite these limitations, to our knowledge, this is the first study to show that the assessment of *ITGAM* SNP (-323G>A) may be a useful marker in assessing the risk of nutritional disorders in patients with HNC undergoing RT. Described limitations (including mainly small sample size) do not allow indisputable conclusions to be drawn and our results should be further investigated in a larger study group for confirmation.

## 5. Conclusions

Determination of the *ITGAM* SNP (-323G>A) may be a useful marker in the assessment of the risk of nutritional disorders in patients with HNC undergoing RT.

## Figures and Tables

**Table 1:** General characteristics of the study group.

Factor		Study Group (*n* = 69)
**Gender**	**Male**	59 (85.51%)
	**Female**	10 (14.49%)
**Age [years]**	Mean ± standard deviation, median (range)	63.5 ± 9.3, 63 (42–87)
	**≥65**	29 (42.03%)
	**<65**	40 (57.97%)
**Histopathological diagnosis**	**Squamous cell carcinoma**	64 (92.75%)
	**Other**	5 (7.25%)
**Tumor location**	**Oropharyngeal**	29 (42.03%)
	**Larynx**	36 (52.17%)
	**Others**	4 (5.80%)
**T stage**	**T1**	2 (2.90%)
	**T2**	12 (17.39%)
	**T3**	22 (31.88%)
	**T4**	33 (47.83%)
**N stage**	**N0**	23 (33.33%)
	**N1**	8 (11.59%)
	**N2**	32 (46.38%)
	**N3**	6 (8.70%)
**M stage**	**M0**	68 (98.55%)
	**M1**	1 (1.45%)
**Disease stage**	**III**	20 (28.98%)
	**IVA**	39 (56.52%)
	**IVB**	4 (5.80%)
	**IVC**	6 (8.70%)
**Performance status (PS)**	**≤1**	59 (85.51%)
	**>1**	10 (14.49%)
**Type of treatment**	**Surgery + RT**	29 (42.03%)
	**Surgery + C-RT**	18 (26.09%)
	**RTH alone**	9 (13.04%)
	**Induction CTH +RT**	3 (4.35%)
	**C-RT**	6 (8.69%)
	**Induction CTH +C-RT**	3 (4.35%)
	**Induction CTH + Surgery + C-RT**	1 (1.45%)
**Alcohol consumption**	**Yes**	29 (42.03%)
	**No**	40 (57.97%)
**Smoking status**	**Smoker**	51 (73.91%)
	**Non-smoker**	18 (26.09%)
	**Current smoker**	47 (92.16%)
	**Former smoker**	4 (7.84%)
	**Smoking during treatment**	43 (91.49%)
	**Does not smoke during treatment**	4 (8.51%)
**Parenteral nutrition**	**Yes**	8 (11.59%)
	**No**	61 (88.41%)
**Weight** [kg]	Mean ± standard deviation, median (range)	65.2 ± 11.4, 66 (43–9)
**BMI** [kg/m^2^]	Mean ± standard deviation, median (range)	23 ± 4,3, 22.8 (14.5–34.4)
**SGA**	**A**	15 (21.74%)
	**B**	32 (46.38%)
	**C**	22 (31.88%)
**NRS**	**2**	47 (68.12%)
	**3**	19 (27.54%)
	**4**	2 (2.90%)
	**5**	1 (1.44%)
**CWL**	**Yes**	25 (36.23%)
	**No**	44 (63.77%)

**Table 2:** Impact of the clinical-demographic, nutritional and genetic factors on the subjective global assessment (SGA) score.

Variable		SGA					
		A	B or C	*p* OR [95%CI]	A or B	C	*p* OR [95%CI]
**Gender**	**Male**	11 (18.64%)	48 (81.36%)	0.1418	39 (66.10%)	20 (33.90%)	0.3907
	**Female**	4 (40.00%)	6 (60.00%)	0.34 [0.08–1.43]	8 (80.00%)	2 (20.00%)	0.49 [0.09–2.51]
**Age (years)**	**≥65**	9 (31.03%)	20 (68.67%)	0.1173	18 (62.07%)	11 (37.93%)	0.3603
	**<65**	6 (15.00%)	34 (85.00%)	2.55 [0.79–8.23]	29 (72.50%)	11 (27.50%)	0.62 [0.22–1.72]
**Histopathological diagnosis**	**Squamous-cell carcinoma**	12 (18.75%)	52 (81.25%)	0.0530	42 (65.63%)	22 (37.37%)	0.2401
	**Others**	3 (60.00%)	2 (40.00%)	0.15 [0.02–1.02]	5 (100.00%)	-	0.17 [0.01–3.25]
**Tumor location**	**Oropharyngeal**	11 (37.93%)	18 (62.07%)	0.0089 *	20 (68.97%)	9 (31.03%)	0.8974
	**Others**	4 (10.00%)	36 (90.00%)	5.50 [1.53–19.71]	27 (67.50%)	13 (32.50%)	1.07 [0.38–2.99]
	**Larynx**	4 (11.11%)	32 (88.89%)	0.0319 *	24 (66.67%)	12 (33.33%)	0.7874
	**Others**	11 (33.33%)	22 (66.67%)	0.25 [0.07–0.89]	23 (69.70%)	10 (30.30%)	0.87 [0.31–2.40]
**T stage**	**T1–3**	15 (41.67%)	21 (58.33%)	0.0080 *	30 (83.33%)	6 (16.67%)	0.0063 *
	**T4**	-	33 (100.00%)	48.30 [2.75–850.05]	17 (51.52%)	16 (48.48%)	4.71 [1.55–14.29]
**N stage**	**N0**	8 (34.78%)	15 (65.22%)	0.0696	19 (82.61%)	4 (17.39%)	0.0753
	**N1–3**	7 (15.22%)	39 (84.78%)	2.97 [0.92–9.63]	28 (60.87%)	18 (39.13%)	3.05 [0.89–10.44]
**M stage**	**M0**	15 (22.06%)	53 (77.94%)	0.9326	47 (69.12%)	21 30.88%)	0.2527
	**M1**	-	1 (100.00%)	0.87 [0.03–22.42]	-	1 (100.00%)	6.63 [0.26–169.40]
**Disease stage**	**III**	7 (35.00%)	13 (65.00%)	0.0948	16 (80.00%)	4 (20.00%)	0.1829
	**IVA-IVC**	8 (16.33%)	41 (83.67%)	2.76 [0.84–9.08]	31 (63.26%)	18 (36.73%)	2.32 [0.67–8.03]
**Performance status (PS)**	**≤1**	12 (20.34%)	47 (79.66%)	0.4967	42 (71.19%)	17 (28.81%)	0.1929
	**>1**	3 (30.00%)	7 (70.00%)	0.60 [0.13–2,65]	5 (50.00%)	5 (50.00%)	2.47 [0.63–9.64]
**Alcohol consumption**	**Yes**	5 (17.24%)	24 (82.76%)	0.4428	16 (55.17%)	13 (44.83%)	0.0530
	**No**	10 (25.00%)	30 (75.00%)	0.62 [0.19–2.08]	31 (77.50%)	9 (22.50%)	0.36 [0.13–1.01]
**Smoking status**	**Smoker**	8 (15.69%)	43 (84.31%)	0.0466 *	32 (62.75%)	19 (37.25%)	0.1178
	**Non-smoker**	7 (38.89%)	11 (61.11%)	2.29 [0.09–0.98]	15 (83.33%)	3 (16.67%)	0.34 [0.09–1.32]
**Concurrent C-RT**	**Yes**	8 (28.57%)	20 (71.43%)	0.4967	19 (67.86%)	9 (32.14%)	0.9696
	**No**	7 (17.07%)	34 (82.93%)	1.94 [0.61–6.17]	28 (68.29%)	13 (31.71%)	0.98 [0.35–2.75]
***ITGAM* genotype**	**AA**	11 (37.93%)	18 (62.07%)	0.0089 *	28 (96.55%)	1 (3.45%)	0.0013 *
	**GA or GG**	4 (10.00%)	36 (90.00%)	5.50 [1.53–19.71]	19 (47.50%)	21 (52.50%)	30.95 [3.83–249.96]
	**GG**	-	6 (100.00%)	0.3412	1 (16.67%)	5 (83.33%)	0.0213 *
	**AA or GA**	15 (23.81%)	48 (76.19%)	0.24 [0.01–4.52]	46 (73.02%)	17 (26.98%)	0.07 [0.01–0.68]

*-statistically significant results.

**Table 3:** Impact of the clinical-demographic, nutritional and genetic factors on the nutrition risk screening (NRS).

Variable		NRS		
		<3	≥3	*p* OR [95%CI]
**Gender**	**Male**	40 (67.80%)	19 (32.20%)	0.8901
	**Female**	7 (70.00%)	3 (30.00%)	0.90 [0.21–3.88]
**Age (years)**	**≥65**	19 (65.52%)	10 (34.48%)	0.6935
	**<65**	28 (70.00%)	12 (30.00%)	0.81 [0.29–2.26]
**Histopathological diagnosis**	**Squamous-cell carcinoma**	45 (70.31%)	19 (29.69%)	0.1834
	**Others**	2 (40.00%)	3 (60.00%)	3.55 [0.55–23.00]
**Tumor location**	**Oropharyngeal**	21 (72.41%)	8 (27.59%)	0.5150
	**Others**	26 (65.00%)	14 (35.00%)	1.41 [0.50–4.01]
	**Larynx**	23 (63.89%)	13 (36.11%)	0.4325
	**Others**	24 (72.73%)	9 (27.27%)	0.66 [0.24–1.84]
**T stage**	**T1–3**	24 (66.67%)	12 (33.33%)	0.7874
	**T4**	23 (69.70%)	10 (59.30%)	0.87 [0.31–2.40]
**N stage**	**N0**	13 (56.52%)	10 (43.48%)	0.1477
	**N1–3**	34 (33.26%)	12 (66.74%)	0.46 [0.16–1.32]
**M stage**	**M0**	46 (67.65%)	22 (32.35%)	0.8216
	**M1**	1 (100.00%)	-	0.69 [0.03–17.59]
**Disease stage**	**III**	12 (60.00%)	8 (40.00%)	0.3576
	**IVA-IVC**	35 (71.43%)	14 (28.57%)	0.60 [0.20–1.78]
**Performance status (PS)**	**≤1**	39 (66.10%)	20 (13.90%)	0.3907
	**>1**	8 (80.00%)	2 (20.00%)	0.49 [0.09–2.51]
**Alcohol consumption**	**Yes**	19 (65.52%)	10 (34.48%)	0.6935
	**No**	28 (70.00%)	12 (30.00%)	0.81 [0.29–2.26]
**Smoking status**	**Smoker**	35 (68.63%)	16 (31.27%)	0.8700
	**Non-smoker**	12 (66.67%)	6 (33.33%)	1.09 [0.34–3.43]
**Concurrent C-RT**	**Yes**	17 (60.71%)	11(39.29%)	0.2779
	**No**	30 (73.17%)	11 (26.83%)	0.57 [0.20–1.58]
***ITGAM* genotype**	**AA**	20 (68.97%)	9 (31.03%)	0.8974
	**GA or GG**	27 (67.50%)	13 (32.50%)	1.07 [0.38–2.99]
	**GG**	4 (66.67%)	2 (33.33%)	0.9365
	**GA or AA**	43 (68.25%)	20 (31.75%)	0.93 [0.16–5.51]

**Table 4:** Impact of the clinical-demographic, nutritional and genetic factors on the critical weight loss (CWL).

Variable		Critical Weight Loss (CWL)		
		No	Yes	*p* OR [95%CI]
**Gender**	**Male**	38 (64.41%)	21 (35.59%)	0.7888
	**Female**	6 (60.00%)	4 (40.00%)	1.21 [0.31–4.76]
**Age (years)**	**≥65**	18 (62.07%)	11 (37.93%)	0.8026
	**<65**	26 (65.00%)	14 (35.00%)	0.88 [0.33–3.38]
**Tumor location**	**Oropharyngeal**	10 (34.48%)	19 (65.52%)	0.0001 *
	**Others**	34 (85.00%)	6 (15.00%)	0.09 [0.03–0.29]
	**Larynx**	32 (88.89%)	4 (11.11%)	<0.0001 *
	**Others**	12 (36.36%)	21 (63.64%)	14 [4.98–49.28]
**Histopathological diagnosis**	**Squamous-cell carcinoma**	42 (65.63%)	22 (34.38%)	0.2681
	**Others**	2 (40.00%)	3 (60.00%)	2.86 [0.44–18.43]
**T stage**	**T1–3**	24 (66.67%)	12 (33.33%)	0.6012
	**T4**	20 (60.61%)	13 (39.39%)	1.30 [0.49–3.48]
**N stage**	**N0**	14 (60.87%)	9 (39.13%)	0.7233
	**N1–3**	30 (65.22%)	16 (34.78%)	0.83 [0.29–2.33]
**M stage**	**M0**	43 (63.25%)	25 (36.75%)	0.7325
	**M1**	1 (100.00%)	-	0.57 [0.02–14.49]
**Disease stage**	**III**	13 (65.00%)	7 (35.00%)	0.8918
	**IVA-IVC**	31 (63.26%)	18 (36.74%)	1.08 [0.36–3.20]
**Performance status (PS)**	**≤1**	37 (62.71%)	22 (37.29%)	0.6585
	**>1**	7 (70.00%)	3 (30.00%)	0.72 [0.17–3.08]
**Alcohol consumption**	**Yes**	19 (65.52%)	10 (34.48%)	0.7969
	**No**	25 (62.50%)	15 (37.50%)	1.14 [0.42–3.09]
**Smoking status**	**Smoker**	29 (56.86%)	22 (43.14%)	0.0543
	**Non-smoker**	15 (83.33%)	3 (16.67%)	0.26 [0.07–1.02]
**Concurrent C-RT**	**Yes**	18 (64.29%)	10 (35.71%)	0.9411
	**No**	26 (63.41%)	15 (36.59%)	1.04 [0.38–2.82]
**NRS**	**<3**	27 (57.45%)	20 (42.55%)	0.1163
	**≥3**	17 (77.27%)	5 (22.73%)	0.39 [0.12–1.26]
**SGA**	**A**	8 (53.33%)	7 (46.67%)	0.3450
	**B or C**	36 (66.67%)	18 (33.33%)	0.57 [0.18–1.83]
	**A or B**	31 (65.96%)	16 (34.04%)	0.5808
	**C**	13 (59.09%)	9 (40.91%)	1.34 [0.47–3.80]
***ITGAM* genotype**	**AA**	21 (72.41%)	8 (27.59%)	0.2062
	**GA or GG**	23 (57.50%)	17 (42.50%)	1.94 [0.69–5.42]
	**GG**	1 (16.67%)	5 (83.33%)	0.0353 *
	**GA or AA**	43 (68.25%)	20 (31.75%)	0.09 [0.01–0.85]

*-statistically significant results.

**Table 5:** Factors affecting overall survival.

Factor	Log-Rank Test			
	Univariate Analysis		Multivariate Analysis ^#^	
	HR [95%CI]	*p*	HR [95%CI]	*p*
**Gender (male)**	1.35 [0.58–3.11]	0.4710	1.18 [0.41–3.40]	0.7599
**Age (≥65 years)**	1.92 [0.99–3.71]	0.0225 *	1.62 [0.83–3.17]	0.1618
**Smoking history (yes)**	0.69 [0.34–1.40]	0.2290	0.66 [0.32–1.36]	0.2631
**Smoking during treatment (yes)**	1.50 [0.28–7.91]	0.6838	0.85 [0.42–1.72]	0.6572
**Alcohol consumption (yes)**	0.78 [0.42–1.44]	0.4063	0.79 [0.41–1.54]	0.4972
**Performance status (>0)**	1.83 [0.71–4.75]	0.1029	1.05 [0.39–2.80]	0.9283
**Tumor location (oropharyngeal)**	1.05 [0.56–1.94]	0.8787	0.98 [0.50–1.94]	0.9587
**Tumor location (larynx)**	0.93 [0.51–1.70]	0.7967	0.95 [0.50–1.83]	0.8860
**T stage (T4)**	1.21 [0.66–2.23]	0.5045	0.95 [0.48–1.86]	0.8714
**N stage (N1–3)**	1.15 [0.62–2.16]	0.6391	1.11 [0.54–2.26]	0.7781
**M stage (M1)**	17.19 [0.01–53382.20]	0.0001 *	6.03 [0.52–69.64]	0.1520
**TNM stage (IV)**	5.82 [0.93–36.55]	<0.0001 *	4.14 [1.35–12.73]	0.0135 *
**Parenteral nutrition (yes)**	0.30 [0.06–1.36]	0.1185	1.81 [0.49–6.73]	0.3801
**Treatment (concurrent C-RT)**	1.00 [0.45–2.26]	0.9905	1.09 [0.48–2.47]	0.8426
**SGA (C)**	0.94 [0.50–1.76]	0.8349	0.46 [0.21–1.02]	0.0562
**SGA (BC)**	1.16 [0.56–2.43]	0.6901	0.95 [0.43–2.14]	0.9097
**NRS (≥3)**	1.51 [0.74–3.10]	0.1833	1.74 [0.84–3.58]	0.1355
**CWL (yes)**	0.94 [0.49–1.80]	0.8539	0.80 [0.38–1.68]	0.5578
***ITGAM* genotype (AA)**	1.16 [0.61–2.19]	0.6106	1.56 [0.75–3.23]	0.2382
***ITGAM* genotype (GG)**	3.34 [0.70–15.97]	0.0056*	3.01 [1.07–8.48]	0.0376 *

^#^-adjusted for statistically significant variables from univariate analysis. *-statistically significant results.

## Supplementary Materials

The following are available online at https://www.mdpi.com/2077-0383/9/12/4041/s1, Table S1. Distribution of demographic, clinical and nutritional variable saccording to ITGAM genotypes. Table S2. Distribution of demographic, clinical and nutritional variables according to SGA. Table S3. Distribution of demographic, clinical and nutritional variables according to NRS and CWL.
