# Supplementary material for: Polymorphism of The Regulatory Region of the ITGAM Gene (-323G>A) as a Novel Predictor of a Poor Nutritional Status in Head and Neck Cancer Patients Subjected to Intensity-Modulated Radiation Therapy
Source: J Clin Med. 2020 Dec 14;9(12):4041. doi: 10.3390/jcm9124041 (PMC7765043; doi:10.3390/jcm9124041)
Supplement: Supplementary file 1 [file jcm-09-04041-s001.pdf]

Table S1. Distribution of demographic, clinical and nutritional variable saccording to *ITGAM* genotypes.

Variable	*ITGAM* genotype median (interquartile range)					
	AA	GA or GG	*p*	GG	GA or AA	*p*
**Age** [years]	64.0 (56.0–76.0)	61.5 (57.0–66.5)	0.3814	65.0 (59.0–69.0)	62.0 (56.0–70.0)	0.7656
**Weight** [kg]	64.0 (53.0–74.0)	66.0 (55.0–73.0)	0.7891	62.5 (54.0–80.0)	66.0 (55.0–74.0)	0.9321
**BMI** [kg/m^2]	24.5 (19.7–25.7)	22.8 (19.8–24.6)	0.3619	21.5 (20.3–22.8)	22.9 (19.7–25.3)	0.5229
**TP** [g/L]	6.6 (6.3–6.9)	6.7 (6.2–7.1)	0.6705	6.8 (6.2–7.1)	6.7 (6.3–7.0)	0.8148
**Albumin** [g/L]	3.4 (3.3–3.7)	3.3 (3.1–3.5)	0.0241 *	3.1 (3.1–3.2)	3.3 (3.2–3.5)	0.0268 *
**Prealbumin** [g/dL]	0.2 (0.2–0.2)	0.2 (0.2–0.3)	0.5473	0.2 (0.2–0.3)	0.2 (0.2–0.3)	0.8899
**Transferrin** [g/L]	2.2 (1.8–2.7)	2.5 (2.1–3.1)	0.2432	2.7 (2.1–3.1)	2.5 (1.9–3.0)	0.4370
**FM** [kg]	20.0 (16.2–24.9)	16.5 (11.9–23.6)	0.1140	26.2 (22.3–30.7)	18.4 (13.6–23.6)	0.1567
**FM%**	28.4 (24.5–36.1)	24.8 (19.2–34.3)	0.1008	32.1 (29.4–37.4)	26.3 (22.6–34.6)	0.2289
**FFM** [kg]	45.5 (42.7–48.7)	48.6 (45.0–53.3)	0.0780	51.8 (46.0–53.7)	46.0 (44.4–53.1)	0.1832
**FFM%**	71.6 (64.1–75.7)	75.2 (65.9–82.3)	0.1168	67.9 (62.6–70.7)	73.9 (65.3–77.6)	0.1903
**Fat-Free Mass Index (FFMI)**[kg/m^2]	16.5 (14.5–17.0)	16.7 (16.0–18.5)	0.0983	17.7 (16.7–21.0)	16.5 (14.8–18.3)	0.0905
**Normalized Fat-Free Mass Index (nFFMI)** [kg/m^2]	16.7 (15.0–18.2)	17.3 (16.4–18.9)	0.1113	18.6 (17.1–22.2)	17.2 (15.1–18.3)	0.0989

* - statistically significant results.

Table S2. Distribution of demographic, clinical and nutritional variables according to SGA.

Variable	SGA median (interquartile range)					
	A	B or C	*p*	A or B	C	*p*
**Age** [years]	66.0 (54.0–76.0)	61.0 (57.0–67.0)	0.4364	63.0 (56.0–68.0)	63.5 (58.0–71.0)	0.7770
**Weight** [kg]	76.0 (69.0–83.0)	62.0 (54.0–68.0)	0.002 *	68.0 (54.0–76.0)	62.5 (55.0–68.0)	0.2970
**BMI** [kg/m^2]	25.0 (24.5–30.1)	21.5 (19.2–24.4)	0.0001 *	24.4 (19.8–25.7)	21.2 (19.0–22.8)	0.0234 *
**TP** [g/L]	6.8 (6.6–7.1)	6.7 (6.2–7.0)	0.1692	6.7 (6.3–7.0)	6.7 (6.2–7.1)	0.6156
**Albumin** [g/L]	3.8 (3.4–3.9)	3.3 (3.2–3.4)	<0.0001 *	3.4 (3.3–3.6)	3.2 (3.1–3.4)	0.0011 *
**Prealbumin** [g/dL]	0.2 (0.2–0.2)	0.2 (0.2–0.3)	0.6209	0.2 (0.2–0.3)	0.2 (0.2–0.3)	0.9180
**Transferrin** [g/L]	2.5 (1.8–3.1)	2.5 (2.0–3.0)	0.7655	2.2 (1.9–3.0)	2.7 (2.1–3.1)	0.4398
**FM** [kg]	17.2 (12.4–24.9)	20.0 (16.4–23.3)	0.1831	18.7 (13.9–24.9)	17.6 (11.2–23.5)	0.5323
**FM%**	27.5 (22.0–36.1)	26.7 (24.5–33.8)	0.7655	26.7 (23.7–34.7)	26.8 (18.1–34.6)	0.5323
**FFM** [kg]	47.7 (44.5–53.2)	45.5 (41.1–54.2)	0.5364	45.9 (44.4–53.2)	49.1 (44.5–53.5)	0.5408
**FFM%**	73.9 (66.0–75.7)	72.6 (64.1–80.4)	0.6837	73.5 (65.3–76.5)	73.0 (65.1–82.13)	0.6155
**Fat-Free Mass Index (FFMI)**[kg/m^2]	16.7 (15.7–18.5)	16.4 (14.3–16.7)	0.0596	16.5 (14.6–18.3)	16.7 (15.4–18.3)	0.5588
**Normalized Fat-Free Mass Index (nFFMI)** [kg/m^2]	17.4 (15.9–19.0)	16.4 (14.8–17.4)	0.0275 *	17.2 (15.1–18.6)	17.2 (15.8–18.6)	0.5408

* - statistically significant results.

Table S3. Distribution of demographic, clinical and nutritional variables according to NRS and CWL.

Variable	NRS median (interquartile range)			CWL median (interquartile range)		
	<3	≥3	*p*	No	Yes	*p*
**Age** [years]	61.0 (56.0–70.0)	63.5 (58.0–66.0)	0.7329	64.0 (57.0–67.0)	61.0 (56.0–70.0)	0.8368
**Weight** [kg]	68.0 (57.0–76.0)	59.5 (51.0–67.0)	0.0208 *	69.0 (64.0–76.0)	61.5 (54.0–69.0)	0.0400 *
**BMI [**kg/m^2**]**	23.1 (20.3–25.7)	19.7 (18.3–23.5)	0.0026 *	22.9 (21.9–25.0)	22.7 (19.6–25.2)	0.3687
**TP** [g/L]	6.7 (6.3–7.0)	6.7 (6.2–7.1)	0.9026	6.7 (6.3–7.1)	6.6 (6.2–6.9)	0.2612
**Albumin** [g/L]	3.3 (3.2–3.5)	3.4 (3.3–3.6)	0.2387	3.3 (3.2–3.5)	3.3 (3.2–3.5)	0.7836
**Prealbumin**[g/dL]	0.2 (0.2–0.3)	0.2 (0.2–0.3)	0.4322	0.2 (0.2–0.2)	0.2 (0.2–0.3)	0.7267
**Transferrin** [g/L]	2.5 (2.0–3.1)	2.2 (1.8–2.7)	0.1605	2.2 (2.0–2.7)	2.6 (2.0–3.1)	0.3589
**FM** [kg]	20.0 (14.2–30.0)	16.3 (11.9–23.3)	0.0833	19.3 (14.4–25.1)	17.2 (11.5–23.6)	0.2747
**FM%**	26.9 (23.7–34.7)	26.5 (22.0–34.6)	0.7524	25.2 (23.7–33.4)	26.8 (22.0–35.4)	0.9751
**FFM** [kg]	47.9 (45.0–53.5)	45.5 (42.9–51.3)	0.1869	53.1 (45.5–54.2)	45.6 (44.5–50.3)	0.0123 *
**FFM%**	73.2 (65.3–76.5)	73.9 (65.2–82.5)	0.4990	74.8 (66.6–76.5)	73.4 (64.6–81.5)	0.8760
**Fat-Free Mass Index (FFMI)**[kg/m^2]	16.7 (16.0–18.5)	16.1 (14.5–16.8)	0.0353 *	17.3 (16.1–18.6)	16.5 (14.7–16.8)	0.0206 *
**Normalized Fat-Free Mass Index (nFFMI)** [kg/m^2]	17.4 (16.4–18.8)	16.5 (14.7–17.8)	0.0291 *	18.5 (16.4–19.7)	17.1 (15.1–17.6)	0.0425 *

* - statistically significant results.
